# De novo electrocardiographic abnormalities in persons living with HIV

**DOI:** 10.1038/s41598-021-00290-x

**Published:** 2021-10-21

**Authors:** Andreas D. Knudsen, Claus Graff, Jonas Bille Nielsen, Magda Teresa Thomsen, Julie Høgh, Thomas Benfield, Jan Gerstoft, Lars Køber, Klaus F. Kofoed, Susanne D. Nielsen

**Affiliations:** 1 Viro-immunology Research Unit, Department of Infectious Diseases 8632, Copenhagen University Hospital, Rigshospitalet, University of Copenhagen, Blegdamsvej 9B, 2100 Copenhagen, Denmark; 2 Department of Cardiology, The Heart Center, Rigshospitalet, University of Copenhagen, Copenhagen, Denmark; 3 Department of Health Science and Technology, Aalborg University, Aalborg, Denmark; 4 Department of Infectious Diseases, Hvidovre Hospital, University of Copenhagen, Hvidovre, Denmark; 5 Department of Radiology, Rigshospitalet, University of Copenhagen, Copenhagen, Denmark

**Keywords:** Epidemiology, Outcomes research

## Abstract

Persons living with HIV (PLWH) may have increased incidence of cardiovascular events and longer QTc intervals than uninfected persons. We aimed to investigate the incidence and risk factors of de novo major electrocardiogram (ECG) abnormalities and QTc prolongation in well-treated PLWH. We included virologically suppressed PLWH without major ECG abnormalities, who attended the 2-year follow-up in the Copenhagen comorbidity in HIV infection (COCOMO) study. ECGs were categorized according to Minnesota Code Manual. We defined de novo major ECG abnormalities as new major Minnesota Code Manual abnormalities. Prolonged QTc was defined as QTc > 460 ms in females and QTc > 450 ms in males. Of 667 PLWH without major ECG abnormalities at baseline, 34 (5%) developed de novo major ECG abnormalities after a median of 2.3 years. After adjustment, age (RR: 1.57 [1.08–2.28] per decade older), being underweight (RR: 5.79 [1.70–19.71]), current smoking (RR: 2.34 [1.06–5.16]), diabetes (RR: 3.89 [1.72–8.80]) and protease inhibitor use (RR: 2.45 [1.27–4.74) were associated with higher risk of getting de novo major ECG abnormalities. Of PLWH without prolonged QTc at baseline, only 11 (1.6%) participants developed de novo prolonged QTc. Five percent of well-treated PLWH acquired de novo major ECG abnormalities and protease inhibitor use was associated with more than twice the risk of de novo major ECG abnormalities. De novo prolonged QTc was rare and did not seem to constitute a problem in well-treated PLWH.

## Introduction

Persons living with HIV (PLWH) have increased risk of cardiovascular disease (CVD), and the rate of sudden cardiac death in PLWH may be as high as 4.5 times of that expected in the uninfected population^1,2^. The resting surface electrocardiogram (ECG) is a widely used tool for assessing and diagnosing cardiac disease, and incident or persistent resting ECG abnormalities are associated with higher risk of subsequent CVD^3,4^. We recently reported higher odds of pathological Q-waves and prolonged corrected QT interval (QTc), markers of coronary artery disease and sudden cardiac death, respectively, among PLWH than among uninfected controls^5^, but little is known about incidence of ECG abnormalities in PLWH.

The pathophysiological mechanisms underlying coronary artery disease and QTc prolongation in PLWH are incompletely understood, but traditional risk factors, such as smoking, are thought to contribute. In addition, HIV-related risk factors including chronic inflammation and use of protease inhibitors as well as specific non-nucleoside reverse transcriptase inhibitors, such as efavirenz and rilpivirine, have been associated with ischemic heart disease and QTc prolongation, respectively^6–11^.

Knowledge about incidence, determinants and risk factors of de novo major ECG abnormalities and abnormal repolarization in PLWH may help identify persons at risk who could profit from closer follow-up and more aggressive management.

The aims of our study were to investigate de novo major ECG abnormalities and prolongation of the QTc interval in a population of well-treated PLWH. We furthermore sought to identify predictors of de novo ECG abnormalities. We hypothesised that both traditional and HIV-related variables, specifically efavirenz, rilpivirine and protease inhibitors, would be associated with de novo prolonged QTc interval and de novo major ECG abnormalities.

## Methods

### Study population

Participants were recruited from the Copenhagen Co-morbidity in HIV Infection (COCOMO) Study (NCT02382822). The COCOMO study is a non-interventional cohort study with the aim of assessing the burden and pathogenesis of non-AIDS comorbidities in PLWH^5,12^. Inclusion criteria were a positive HIV test and age older than 18 years. Between March 2015 and December 2016, 1099 participants were enrolled in the study, representing more than 40% of PLWH residing in the Copenhagen area. The procedures for recruitment and data collection have been described in detail elsewhere^12^. All participants provided oral and written informed consent before study inclusion. The COCOMO study (H-8-2014-0004) has been approved by the Ethics Committee of the Capital Region and the Danish Data Protection Agency. All procedures were performed in accordance with relevant guidelines and regulations.

At enrolment, participants answered structured questionnaires to collect information regarding medical history, symptoms, smoking, alcohol consumption and use of medication. Furthermore, a physical exam including anthropometrics and blood pressure measurements was performed. All clinical examinations were performed by trained clinical staff. Low density lipoprotein (LDL) was collected from blood samples. HIV-related characteristics including mode of transmission, HIV-RNA, CD4 T cell count, CD4 nadir, time since HIV diagnosis, history of AIDS, history of antiretroviral therapy, and use of Methadone were obtained through review of the patients’ medical records. All participants were offered to have a 12-lead resting ECG recorded. Between March 2017 and December 2018, all COCOMO participants were invited to attend a two-year follow-up examination with repeat physical exam, questionnaire, and a 12-lead ECG. A review of medical records for signs of cardiac events between study entry and December 2018, at which time inclusion for two-year follow-up ended, were conducted. For this study, we included participants with suppressed viral load (< 50 copies/mL) at both baseline and follow-up, an available ECG from both baseline and from the two-year follow-up, and no major ECG abnormalities at baseline.

### Definitions

Demographic information was defined according to status at enrolment. We defined hypertension in accordance with the International Society of Hypertension and European guidelines, as current anti-hypertensive treatment and/or systolic blood pressure ≥ 140 mmHg and/or diastolic blood pressure ≥ 90 mmHg at enrolment^13,14^. Body mass index (BMI) was defined according to the WHO classification (< 18.5 underweight, 18.5–24.99 normal weight, 25–29.99 overweight and ≥ 30 kg/m^2^ obese). Heavy drinking was defined according to The National Institute on Alcohol Abuse and Alcoholism (NIAAA) in the United States’ definition as more than 14 standard drinks per week on average (one drink is defined as 14 g of alcohol)^15^. Marijuana use was defined as self-reported use of marijuana. Monthly use of recreational drugs was defined as self-reported use of recreational drugs other than alcohol and marijuana (e.g. Cocaine, ecstasy, heroin, benzodiazepine, amphetamine) more than once a month on average. Low CD4 cell count nadir was defined as below 200 cells/µL. Protease inhibitor use was defined as use of one or more HIV protease inhibitors (class stem *-navir*) at baseline. Methadone status was dichotomised as yes/no at baseline and at follow up. As there were no changes in methadone status among the participants, methadone status was collapsed into a single variable. Cardiac events were defined according to the International Classification of Diseases v10 list of cardiac diagnoses (ICD-10) as one or more of the following: I20-I25 (ischaemic heart diseases) or I30-I52 (other forms of heart disease). Cardiac events were recorded from medical records if they occurred between baseline and end of two-year follow-up.

### ECG recording

ECGs were recorded at baseline and after two years. At both baseline and two-year follow-up, ECGs were recorded by trained medical professionals at Rigshospitalet University Hospital using a CardioSoft electrocardiograph Module and CardioSoft v6.7 Diagnostic System (GE Healthcare) software. Participants were placed in supine position and instructed not to speak or make any movements while recording. The operator allowed the software one minute to calibrate. A retrospective resting ECG of the preceding 10 s was only recorded and stored when all lead readings were calibrated, and no preventable muscular interference was visible.

Recorded ECGs were transferred to the MUSE Cardiology Information System (GE Healthcare, Wauwatosa, WI) (pseudo anonymized) in standard 12-lead format. In case of multiple stored ECGs on one examination day, the last ECG was used.

### Electrocardiographic outcomes

ECGs were coded according to the Minnesota code manual for electrocardiographic findings (MC) and the validated Marquette 12SL algorithm (Software version 243)^16^. The algorithm defines a median complex for each lead and a global median complex for each ECG. Criteria for major MC ECG abnormalities were any of the following (see also supplement 1): Major Q-wave abnormalities (MC 1.1 or 1.2); Minor Q-wave abnormalities plus ST-T abnormalities (MC 1.3 plus MC 4.1 or 4.2, or 5.1 or 5.2); Major Isolated ST-T abnormalities (MC 4.1 or 4.2 or 5.1 or 5.2); intraventricular block (MC 7.1 or 7.2 or 7.4); Right bundle branch block with left anterior hemiblock (MC 7.8); Left ventricular hypertrophy plus ST-T abnormalities (MC 3.1 plus MC 4.1 or 4.2 or 5.1 or 5.2); Major QT prolongation (QTI ≥ 116%); Atrial Fibrillation or Flutter (MC 8.3); Major AV conduction abnormalities including third-degree AV block (MC 6-1), second-degree AV block (MC6.2) and ventricular preexcitation pattern (Wolff-Parkinson-White) (MC 6.4); Other major arrhythmias (MC 8.2). Pathologic Q-waves was defined as major Q-wave (MC 1.1 or 1.2). ECGs with one or more major abnormalities were manually overread, as previously described^5^. ECG interval- and segment lengths were measured from the global median complex. The QT-interval was defined as start of Q-wave to end of T-wave and corrected QT-interval (QTc) was calculated using Bazett’s formula (QT/$\surd RR$). We defined prolonged QTc as QTc > 460 ms in females and QTc > 450 ms in males. New prolonged QTc was defined as prolonged QTc at follow up among those who had normal QTc interval at baseline. In a sensitivity analysis, we used Framingham’s formula for QT interval correction, ${QTc}_{Framingham}=QT+0.154 (1-RR)$. Participants with a QRS duration of 120 ms or more or with ventricular rates > 100 bpm, were not included in the analyses of QT interval prolongation. All ECG analyses were performed blinded to participants’ characteristics.

### Statistics

Continues variables are presented as means with standard deviations (SD) and categorical variables are presented as numbers and percentages. We used multivariable Poisson regression to investigate if potential risk factors were associated with de novo ECG abnormalities. We adjusted for a model including age, sex, smoking status, hypertension, BMI, and diabetes, which were selected a priori. In two separate sensitivity analyses, we further adjusted for LDL (continuous) and methadone, respectively. We furthermore tested if the most commonly used antiretrovirals were associated with de novo prolonged QTc interval. When we explored associations between efavirenz or rilpivirine and QTc, we included only participants who used the respective drugs at both baseline and follow-up. Results were presented as crude and adjusted relative risk (RR) with confidence intervals. We interpreted a p-value of 0.05 or less to infer statistical significance. All statistical analyses were conducted using R^17^ with the sandwich and ggplot2 packages^18–20^.

## Results

**Figure 1:**
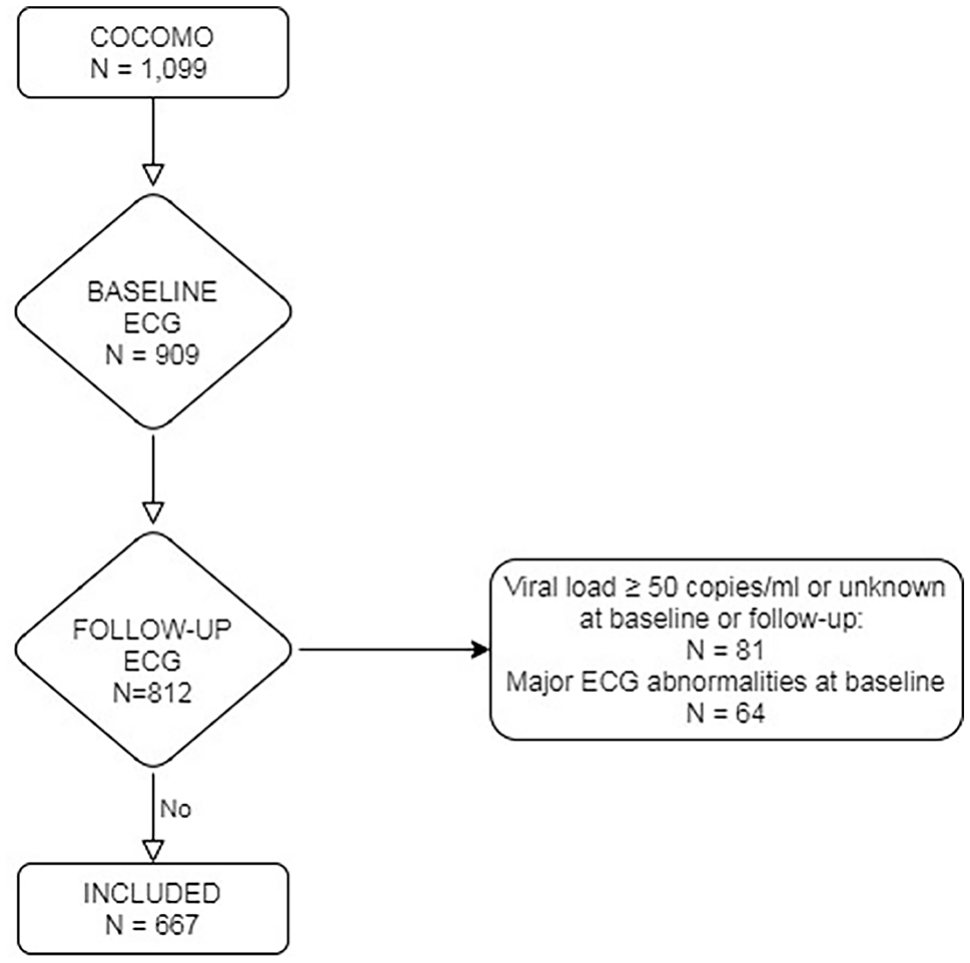
Study overview. The Copenhagen Comorbidity in HIV infection study included 1099 persons living with HIV of which 909 had an electrocardiogram (ECG) measured at baseline. Participants were invited for a repeat exam after two years and 812 of those with a recorded ECG at baseline had a follow-up ECG recorded. Participants with major ECG abnormalities at baseline or participants with viral load ≥ 50 copies/mL were excluded. In total, 667 participants were included in the study.

Of the 1099 participants in COCOMO, 909 (83%) had an ECG recorded at baseline. After a median follow-up of 2.3 years (interquartile range, IQR: 2.1 to 2.4), 812 of these (89%) had a repeat ECG recorded. After excluding participants with viral replication and/or one or more major ECG abnormalities at baseline, 667 participants were eligible for inclusion in the present study (Fig. 1). Mean age at baseline was 51 years (standard deviation, SD: 11) and 570 (85%) were male. Further demographic and clinical characteristics are listed in Table 1.

**Table 1:** Participant characteristics.

	N = 667
Age at baseline (SD)	50.5 (10.7)
Male, n (%)	570 (85)
Smoking, n (%)	
Never smoker	237 (36)
Current smoker	173 (26)
Former smoker	244 (37)
Hypertension, n (%)	284 (43)
Diabetes, n (%)	21 (3.1)
CD4 nadir < 200 cells/µL, n (%)	271 (41)
History of AIDS, n (%)	110 (16)
Protease inhibitor at baseline, n (%)	191 (29)
Protease inhibitor at follow-up, n (%)	175 (26)
Darunavir at baseline, n (%)	111 (17)
Darunavir at follow-up, n (%)	101 (15)
Atazanavir at baseline, n (%)	74 (11)
Atazanavir at follow-up, n (%)	69 (10)
Rilpivirine at baseline, n (%)	24 (3.6)
Rilpivirine at follow-up, n (%)	30 (4.5)
Efavirenz at baseline, n (%)	210 (31)
Efavirenz at follow-up, n (%)	178 (27)
Methadone uses, n (%)	12 (1.8)

### De novo major ECG abnormalities

De novo major ECG abnormalities were present in 34 (5%) participants of whom 6 (18%) had a cardiac event between baseline and follow-up documented in their medical records. Table 2 shows the distribution of ECG abnormalities. In unadjusted analyses, risk of getting any de novo major ECG abnormality at follow-up was associated with higher age (relative risk (RR): 1.59 [95% confidence interval: 1.13, 2.23], per decade older), diabetes (RR: 4.14 [1.6, 10.71]), being underweight (RR: 5.09 [1.61, 16.10]) and baseline protease inhibitor use [RR: 2.22 [1.15, 4.25]).

**Table 2:** De novo major abnormalities at follow-up.

Major abnormalities^‡^ (any), n (%)	34 (5.1)
Major Q wave, n (%)	16 (2.4)
Minor Q wave *plus* ST-T abnormality, n (%)	2 (0.3)
Major isolated ST-T abnormality, n (%)	14 (2.1)
Any intraventricular block, n (%)	9 (1.3)
RBBB with left anterior hemiblock, n(%)	0 (0)
Brugada pattern, n (%)	0
Left ventricular hypertrophy *plus* ST-T abnormality, n (%)	2 (0.3)
Atrial fibrillation/flutter, n (%)	3 (0.4)
Wolf–Parkinson–White, n (%)	1 (0.1)
Prolonged QTc*	11 (1.3)
QTc* > 480 ms	2 (0.3)
QTc* > 500 ms	0 (0.0)

*RBBB* right bundle branch block.

^‡^Any participant may have more than one major abnormality.

*Corrected using Bazett’s formula (QT/√RR).

**Figure 2:**
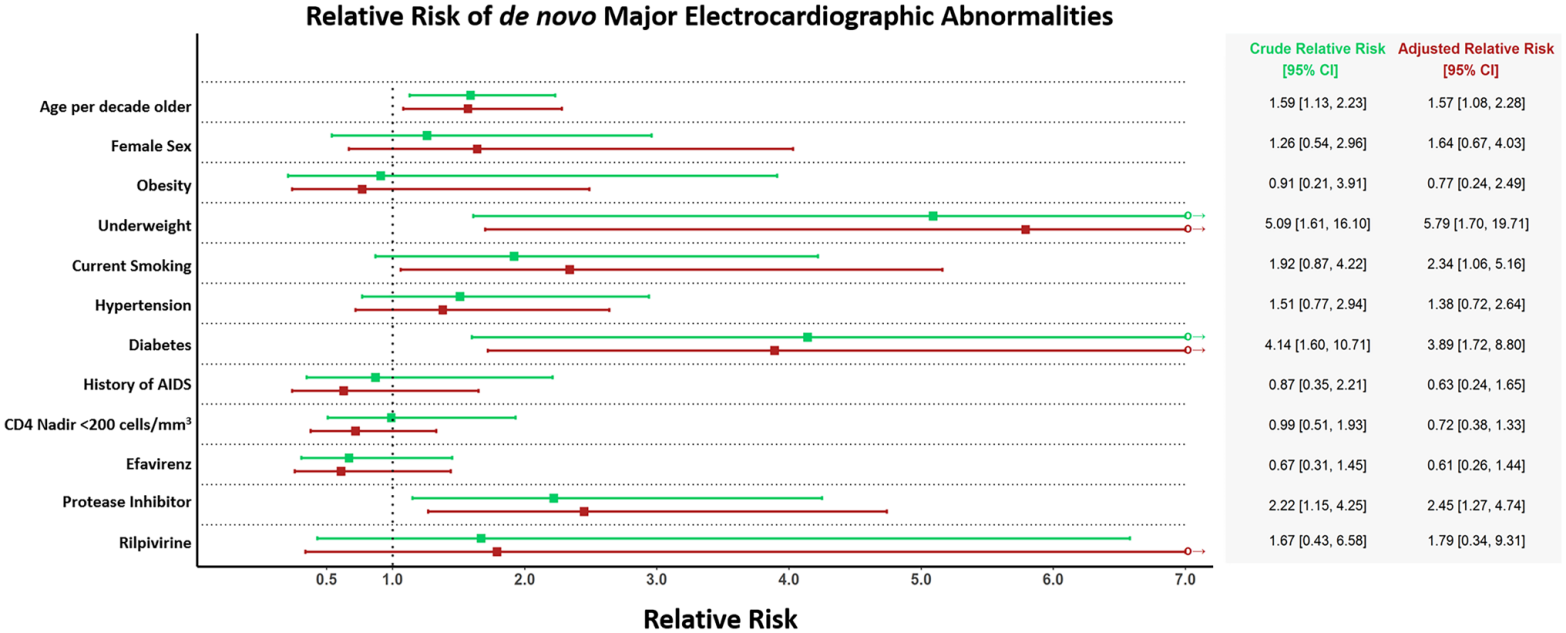
Relative Risk of de novo Major Electrocardiographic Abnormalities. Relative risk (RR) of de novo major Electrocardiographic abnormalities for different predictors with 95% confidence intervals. Vertical dotted line represents an RR of 1. Green is crude (unadjusted) and red is adjusted for age, sex, smoking status, hypertension, BMI, and diabetes. *Age* age at baseline, *obesity* body mass index (BMI) ≥ 30 kg/m^2^, *underweight* BMI < 18.5 kg/m^2^.

In multivariable analyses adjusting for age, sex, smoking status, hypertension, BMI, and diabetes, age (RR: 1.57 [1.08, 2.28] per decade older), current smoking (RR: 2.34 [1.06, 5.16]), diabetes (RR: 3.89 [1.72, 8.80]) being underweight (RR: 5.79 [1.70, 19.71]) and baseline protease inhibitor use (RR: 2.45 [1.27, 4.74]) were associated with higher risk of getting any de novo major ECG abnormality at follow-up. Darunavir and atazanavir, the two most commonly used protease inhibitors, were associated with a RR of any de novo major ECG abnormality of 1.67 [0.83, 3.34], p = 0.151 and 2.34 [0.98, 5.60], p = 0.056, respectively (p for difference = 0.486). There was little evidence for associations between other variables and de novo major ECG abnormalities (Fig. 2). No specific antiretroviral drug class was associated with de novo major ECG abnormalities and CD4 count was not associated with de novo major ECG abnormalities (supplemental Fig. 1).In a sensitivity analysis, we examined whether further adjustment for serum LDL concentration would attenuate the association between protease inhibitor use and de novo major ECG abnormality at follow-up. After adjusting for LDL, protease inhibitor use remained associated with RR: 2.41 [1.24, 4.67] of de novo major ECG abnormality, and LDL concentration, too, was associated with de novo major ECG abnormality (RR: 1.36 [1.01, 1.82] per mM). In sensitivity analyses, we excluded participants who changed to or from protease inhibitors (N = 38) from the analyses. In the resulting population subset (N = 629), protease inhibitor use was associated with an RR of de novo major ECG abnormalities of 2.41 [1.24, 4.69].

### QTc interval

**Figure 3:**
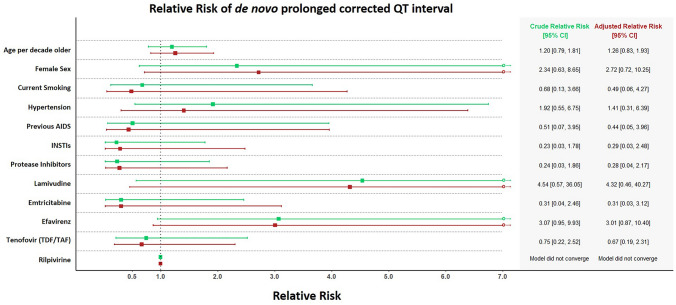
Relative Risk of de novo prolonged corrected QTc. Relative risk (RR) of de novo prolonged corrected QTc for the most commonly used antiretrovirals with 95% confidence intervals. Vertical dotted line represents an RR of 1. Green is crude (unadjusted) and red is adjusted for age, sex, smoking status, hypertension, body mass index (BMI), and diabetes. *Age* age at baseline, *INSTI* integrase nuclear strand transfer inhibitor, *TDF* Tenofovir disoproxil; *TAF* Tenofovir alafenamide.

At baseline, 24 participants had prolonged QTc. Between baseline and follow-up, 11 participants with normal QTc interval at baseline developed de novo prolonged QTc interval according to Bazett’s correction and 9 developed de novo prolonged QTc interval according to Framingham’s correction. Regardless of correction method, we did not find any variables to predict de novo prolonged QTc or QTc interval ≥ 480 ms in univariable or in multivariable analyses (Fig. 3). The crude and adjusted RR for prolonged QTc for efavirenz use were 3.07 [0.95, 9.93], p = 0.061, and 3.01 [0.87, 10.40], p = 0.082, respectively. Methadone use, heavy drinking, marijuana use and monthly use of recreational drugs other than alcohol and marijuana, were not associated with de novo prolonged QTc interval.

## Discussion

In a large cohort of well-treated PLWH, we found five percent of participants to have acquired de novo major ECG abnormalities after a median follow-up of just over two years. Importantly, use of protease inhibitor was associated with more than twice the risk of developing major ECG abnormalities. Few participants developed de novo prolonged QTc, and we did not find any risk factors associated with de novo prolonged QTc although efavirenz use was borderline associated with a higher risk of de novo prolonged QTc.

Little is known about development of de novo major ECG abnormalities in PLWH. Assuming cumulative risk increases linearly over time, our results are comparable with results from the large, community based, *Atherosclerosis Risk in Communities (ARIC)* Study where 8% of similarly aged participants developed de novo Q-waves and/or ST-T abnormalities over a period of three years^21^. However, risk of de novo major ECG abnormalities in our study was four times higher than in a study of patients with type 1 diabetes over the age of 40^22^. As CVD hazard increases with age, comparing populations with different age distributions should be done with caution. In a review of the medical records, we found that just around one in five of participants with de novo major ECG abnormalities had had a cardiac event recorded in their medical records in the follow-up period. This may imply that many cardiac abnormalities are subclinical and remain unnoticed by both patient and treating physician.

Studies consistently report higher risk of myocardial infarction in PLWH than in uninfected populations^1,2,23^, and de novo Q-waves and/or ST-T abnormalities, both signs of ischaemic injury and predictors of major adverse cardiovascular events^21,24,25^, were, indeed, the most common de novo major ECG abnormalities in our cohort. Protease inhibitors have been reported to affect lipid metabolism^26–30^, and protease inhibitor use has been linked to increased risk of CVD in several cohort studies^6–8,31,32^. The more than twofold higher risk of getting de novo major ECG abnormalities among those who used protease inhibitors is similar to the risk of myocardial infarction associated with protease inhibitors^8^. We did not find any individual protease inhibitor to convey a higher risk than others and no individual protease inhibitor was significantly associated with higher risk of de novo major ECG abnormality although the association with atazanavir was borderline significant. Our results indicate that even contemporary protease inhibitors convey cardiovascular risk, and LDL-dyslipidemia did not seem to mediate the increased risk of de novo major ECG abnormalities associated with protease inhibitor use, as the association was not affected by adjusting for LDL. Similar findings have been presented in the Data Collection on Adverse events of Anti-HIV Drugs (D:A:D) study where adjusting for serum lipid levels attenuated but did not remove the association between protease inhibitor use and CVD^6,31^. Traditional risk factors for CVD, such as age, smoking, and diabetes, were all predictors of de novo major ECG abnormalities which supports the guideline recommendations for vigilant risk factor management^33^.

Obesity is a well-established risk factor for cardiovascular disease^34^. Surprisingly, we found low BMI to be associated with higher risk of de novo major ECG abnormalities. Overall mortality is increased among those who are underweight^35^, and other studies have described a U-shaped relationship between BMI and cardiovascular risk^36^. Extremes in BMI, such as anorexia nervosa, may influence the ECG through electrolyte derangements^37^, however, low bodyweight may also act as a surrogate for poor overall health or preclinical cardiovascular disease, and the observed association could be due to unmeasured confounding.

Prolongation of the heart rate corrected QT interval is an often-used marker of delayed repolarization. We examined efavirenz, as this drug previously has been associated with QTc interval prolongation and is considered a drug with possible risk of torsade de pointes^9,10,38^. As almost a third of our participants used efavirenz as part of their antiretroviral regimen, an association would be clinically relevant. Our data did give some evidence to suggest an association between efavirenz use and prolonged QTc in well-treated PLWH, both before and after adjustment, but the association did not reach statistical significance according to our prespecified cut-off. Moreover, the parameter estimation for efavirenz was in the same order of magnitude as that of lamivudine. Studies with larger populations samples are needed to explore this further.

Some limitations to our study warrant mentioning. As we wanted to explore de novo major ECG abnormalities, we included only participants with both baseline and follow-up ECGs in our analyses. Thus, selection bias is possible, as choosing to participate in a study might be influenced by general health. Our study was observational, and some clinically relevant ECG abnormalities such as QTc > 500 ms may have been discovered and intervened against by treating physicians whereby they would not appear on our recordings^39^. However, prevalence of QTc > 500 ms is thought to be rare^40^. Moreover, participants who died before their follow-up did not contribute, and we did also not consider other competing risks. In addition, we chose to use variables collected at baseline to predict future changes in ECG outcomes. As some variables, including protease inhibitor use, might change over time, the exact effect size associated with these variables may vary. However, results were consistent in sensitivity analyses where participants who changed from or to protease inhibitors, were excluded. Strengths of our study include the study size and the detailed, standardized data collection including automatic ECG readings—limiting measurement errors due to reader variability—of both men and women with HIV.

## Conclusion

In conclusion, five percent of well-treated PLWH with no major ECG abnormalities acquired de novo major ECG abnormalities after a two-year period and protease inhibitor use was associated with twice the risk of getting major ECG abnormalities. De novo prolonged QTc was rare and did not seem to constitute a problem in PLWH.

## Supplementary Information

Supplementary Information.

Supplementary Figure 1.
