# Supplementary material for: De novo electrocardiographic abnormalities in persons living with HIV
Source: Sci Rep. 2021 Oct 21;11:20750. doi: 10.1038/s41598-021-00290-x (PMC8531322; doi:10.1038/s41598-021-00290-x)
Supplement: Supplementary file 1 — Supplementary Information. [file 41598_2021_290_MOESM1_ESM.docx]

SUPPLEMENTUM [TABLE]

TABLE TITLE: Major Minnesota Code Manual Abnormalities

| **Major Minnesota Code Manual Abnormalities** | |
| --- | --- |
|  | Minnesota Code (MC) |
| Major Q wave abnormalities (Old prevalent MI) | MC 1-1, 1-2 |
| Minor Q wave abnormalities *plus* ST-T abnormalities (Possible old MI) | MC I-3 *plus* MC 4-1 or 4-2, or 5-1 or 5-2 |
| Major Isolated ST-T abnormalities | MC 4-1 or 4-2 or 5-1 or 5-2 |
| Complete or intermittent LBBB | MC 7-1 |
| Complete or intermittent RBBB | MC 7-2 |
| Nonspecific intraventricular block | MC 7-4 |
| RBBB with left anterior hemiblock | MC 7.8 |
| Brugada pattern | MC 7-9 |
| Left ventricular hypertrophy *plus* ST-T abnormalities | MC 3-1 *plus* MC 4-1 or 4-2 or 5-1 or 5-2 |
| Major QT prolongation | QTI ≥ 116% |
| Atrial Fibrillation or Flutter (Continuous or intermittent) | MC 8-3 |
| Major AV conduction abnormalities   - Third-degree AV block (AVB3) - Second-degree AV block (AVB2) - Ventricular preexcitation pattern (WPW) - Artifi cial pacemaker | MC 6-1  MC 6-2  MC 6-4  MC 6-8 |
| Other major arrhythmias  Ventricular fibrillation *or* Ventricular asystole | MC 8-2 |
| Supraventricular tachycardia (SVT) | MC 8-4-2 *or* MC 8-4-1with HR>140 |

Supplemental figure 1

Relative Risk of de novo Major Electrocardiographic Abnormalities for antiretrovirals other than protease inhibitors

Relative risk (RR) of de novo major Electrocardiographic abnormalities for INSTIs, NRTIs, and NNRTIs with 95% confidence intervals. Vertical dotted line represents an RR of 1. Green is crude (unadjusted) and red is adjusted for age, sex, smoking status, hypertension, body mass index (BMI), and diabetes. Age: age at baseline; INSTI: integrase nuclear strand transfer inhibitor; NRTI: Nucleoside/nucleotide reverse-transcriptase inhibitors; NNRTI: Non-nucleoside reverse-transcriptase inhibitors.

**Abbreviations**

ARIC Atherosclerosis Risk in Communities (ARIC) study

BMI Body Mass Index

COCOMO Copenhagen Comorbidity in HIV infection Study

CVD Cardiovascular disease

CYP2B6

ECG Electrocardiogram

HIV Human immunodeficiency viruses

IQR Interquartile range

LDL Low density lipoprotein

MC Minnesota Code

PLWH Persons living with HIV

RBBB Right bundle branch block

RR Relative Risk
