# Supplementary figures and images for: De novo electrocardiographic abnormalities in persons living with HIV
Source: Sci Rep. 2021 Oct 21;11:20750. doi: 10.1038/s41598-021-00290-x (PMC8531322; doi:10.1038/s41598-021-00290-x)

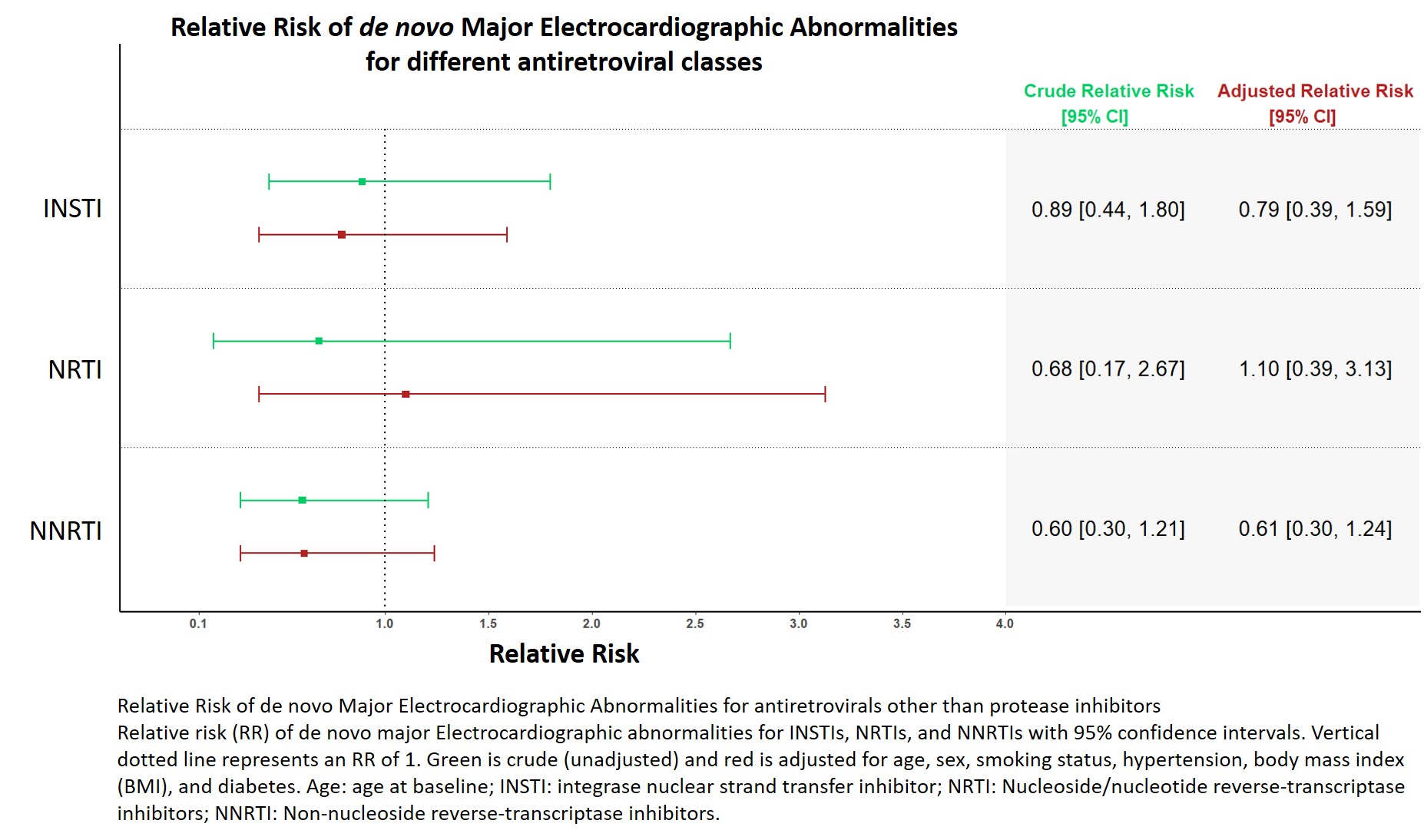

Supplement: Supplementary file 2 — Supplementary Figure 1. [file 41598_2021_290_MOESM2_ESM.jpg]
